# Neuromagnetic representation of vowel prosody in the auditory cortex

**DOI:** 10.1038/s41598-026-68466-x

**Published:** 2026-09-01

**Authors:** Esther Megbel, Kurt Steinmetzger, André Rupp, Martin Andermann

**Affiliations:** 1 Section of Biomagnetism, Department of Neurology, Heidelberg University Hospital, Im Neuenheimer Feld 400, 69120 Heidelberg, Germany; 2 Department of Child and Adolescent Psychiatry and Psychotherapy, Central Institute of Mental Health, J 5, 68159 Mannheim, Germany; 3 Tinnitus Center, Charité - Universitätsmedizin Berlin, Luisenstraße 13, 10117 Berlin, Germany

**Keywords:** Prosody, Vowels, Magnetoencephalography, Auditory cortex, Auditory evoked responses, Neuroscience, Psychology, Psychology

## Abstract

A key feature of vowels in spoken language is that their fundamental frequency (*f*_0_), which underlies perceived pitch, can change dynamically over time. However, it remains elusive how such prosodic glides shape the neural response in the auditory cortex. The current magnetoencephalography (MEG) experiment was designed to study the transient and sustained neuromagnetic correlates of prosodic contour information in vowels, with a particular focus on the P2 component. Twenty-four normal-hearing participants passively listened to vowel sequences in which both vowel type and prosodic contour were either unchanged or varied between successive vowels. At the level of single vowels, the results showed that falling contours elicit larger and earlier P2 responses than rising and flat prosodic contours. At the level of vowel triplets, the P2 was larger in response to stimulus sequences in which prosodic contour was varied across stimuli. The data indicate that the P2 might be a neural correlate of the early processing of prosodic contour information; moreover, they suggest that the P2 might be particularly sensitive to pitch glides, possibly due to longer time constants that are encompassed within the P2.

**Supplementary Information:**

The online version contains supplementary material available at https://doi.org/10.1038/s41598-026-68466-x.

## Introduction

Vowels constitute a central component of syllable structure in spoken language. They contain characteristic formants, i.e., overtone clusters that form when acoustic energy concentrates around a specific frequency region. The relative position of formants within the frequency spectrum distinguishes vowels from one another^1^. While formant frequencies appear comparatively stable over the nucleus of a vowel (^2,3^; however, see^4^), its fundamental frequency (*f*_0_) can vary substantially across time, and time-varying *f*_0_ patterns constitute an important dimension of prosody in speech and melody in music^5^. In the present study, we use the term prosody in a narrow operational sense, referring exclusively to the pitch contour of a vowel (as defined by its *f*_0_) across time, while other prosodic dimensions such as duration, intensity, and voice quality are held constant.

Neural processing of prosody and vowel changes in the auditory cortex can be investigated by means of magnetoencephalography (MEG), a non-invasive method that provides excellent temporal and good spatial resolution^6^. The neuromagnetic response to the onset of periodic sounds has a typical, time-variant pattern, the auditory evoked field (AEF): The first 250–350 ms comprise a tripartite transient response (including P1, N1, and P2) that subsequently evolves into a sustained field (SF). The SF builds up until about 400 ms after sound onset^7^ and persists until sound offset^8,9^; it provides a continuous representation of pitch in the ongoing sound^8–10^.

The early transient AEF are thought to mirror functionally distinct stages of auditory processing but to date, their functional significance in prosody processing is not entirely understood^11,12^. While the P1 wave has been more associated with the registration of basic acoustic features^13,14^, the N1 represents a multi-component complex with particular sensitivity to changes in glottal pulse rate as well as formant position and relation^15^; in contrast, the less-frequently studied P2 is thought to reflect higher-order feature integration^16^, likely including the extraction of relational properties over longer temporal windows. In the context of vowel processing, this might suggest that the time-varying pitch pattern of a prosodic contour is reflected more strongly in later components such as the P2 than in earlier AEF^10,17,18^.

Many neurophysiological studies on cortical pitch processing have focused on the N1 response. Previous work showed that the N1 is sensitive to pitch height^10,19,20^, but pitch contour as in prosodic glides spans over a longer time period that the N1 may not properly reflect^21^. Patterson et al.^17^ compared the neural activity in response to melodic sequences in which pitch was either fixed or varied between successive sounds; here, both the N1 and P2 waves were larger in varying (i.e., melodic) than in fixed pitch sequences^18^, and subsequent studies corroborated the notion that the P2 might play a particular role in melody processing^10^. Measuring the AEF evoked by upward versus downward pitch transitions, Andermann et al.^10^ observed that the P2 provided a representation of relative pitch; more specifically, they showed that P2 responses occurred earlier following upward as compared to downward pitch transition, and that larger pitch shifts were associated with larger P2 responses. Assuming that the P2 represents integration of information over a longer time period than the N1, this finding supports the notion that the P2 might be particularly sensitive to represent pitch glides. In an electroencephalography (EEG) study, Bidelman noted similar findings^21^: Examining the sensitivity of the pitch onset response (POR, a sub-component of the N1), he found that this activity did not distinguish between upward and downward pitch glides. The POR latency was, however, shorter following stimuli with static as compared to dynamic pitch contours. Bidelman inferred that integrating stimuli with time-varying pitch contours might require longer temporal windows.

Although both Andermann et al. and Bidelman used non-speech sounds, their findings might at least in part be transferable to pitch processing in the context of language: As early pitch processing in music and language underlies shared neural paths^22–24^, one would expect the early transient and sustained neural responses to speech sounds to evolve in a similar manner. At the same time, however, little is known about the effects of pitch *glides* which are more common in prosodic contours than the discrete tone steps that make up tonal melodies. In a companion experiment of the current work, we did focus on such prosodic contours^25^. Using functional near-infrared spectroscopy (fNIRS) and EEG simultaneously, Steinmetzger et al.^25^ investigated the cortical response to continuous prosodic contours in natural vowel sequences. They demonstrated that stimulus sequences in which the prosodic contour varied between successive vowels evoked enhanced P2 and SF activity. However, their analysis remained at the level of stimulus sequences and did not include the response to the prosodic contour or vowel type within *single* stimuli, as is the case in the present paper.

There are also several other neurophysiological studies that have investigated the cortical response to prosody in speech^26–28^. For example, Lialiou et al.^26^ recently demonstrated differential attentional orienting responses to rising versus falling intonational contours; moreover, Hsu et al.^27^ reported an interesting EEG experiment in which rising prosodic contours lead to larger P300 waves and decreased right-hemispheric oscillations in the beta-band. The results from those studies are, however, difficult to compare with the above-described findings because they often involve sensor-level analyses of deviant responses (e.g., the mismatch negativity or the P300 wave) that arise within oddball paradigms. In contrast, experiments that assess prosodic contours in single natural vowels, employing equiprobable stimuli and source-level modeling of the early evoked activity, appear to be scarce or absent.

Cortical pitch processing is not only driven by bottom-up responses to incoming stimuli; the auditory system is also thought to provide top-down predictions on how a sound pattern will continue, ensuring that incoming stimuli are processed efficiently^29,30^. Within such a ‘predictive coding’ framework, transient AEF responses can be understood as reflecting prediction error: vowel sequences in which prosodic contour changes between successive vowels (termed varying prosodic contour sequences hereafter) would then be expected to evoke larger AEF (because of their comparably low predictability), while sequences in which prosodic contour does *not* change between successive vowels (termed repeated prosodic contour sequences hereafter) would lead to neural adaptation (i.e., response suppression^31^) of the subsequent AEF, due to their higher predictability^32,33^. Previous MEG research has revealed that fixed pitch sequences elicit decreased N1^34^ and P2 responses^18,35^; the N1 amplitudes additionally decrease in response to familiar melodies^36^. Andermann et al.^10^ provided evidence that P1, N1, and P2 amplitudes decrease in response to fixed pitch sequences, while varying pitch sequences evoked strong AEF responses without decreasing magnitude; this was also the case in response to later sounds in the sequence, i.e., when its fixed vs. varying nature was already known to the listeners, arguing in favor of an early process^10^. Transferred to the context of the present work, one would then assume that if the P2 is involved in the predictive processing of prosodic contours, it should evolve with larger amplitude in varying prosodic contour sequences where prosodic contour varies between successive vowels (low predictability), whereas it should show repetition suppression in repeated prosodic contour sequences where prosodic contour is invariant between the vowels (high predictability).

To sum up, the functional significance of transient and sustained AEF responses in the processing of prosodic contours is not fully understood. The goal of the current MEG experiment was, therefore, to study the neuromagnetic correlates of prosodic contour in vowels, with a particular focus on the P2 response. As the P2 appears to be involved in relative pitch processing and is affected by neural adaptation^10^, we would also expect enhanced P2 activity in response to vowels with dynamic (i.e., rising and falling) as compared to constant (i.e. flat) prosodic contours, and a response decrement in highly-predictable vowel sequences where all vowels have the same prosodic contour (repeated prosodic contour sequences). As a secondary goal of the study, we also wished to analyze the P2 response to single vowels and vowel transitions, in an effort to parallel earlier research^15^ where such transitions were found to elicit prominent N1 responses.

## Material and methods

### Participants

Twenty-four adult volunteers participated in the experiment (14 females; 1 left-handed; mean age: 25.9 years, SD: 7.3 years); they were all native speakers of German. None of the subjects reported any history of hearing impairment; normal hearing was verified with a brief audiometric screening (hearing loss < 20 dB between 125 Hz and 4 kHz). The experimental procedures were approved by the local ethics board (Medical Faculty, University of Heidelberg; study ID: S-378/2018) and were conducted in compliance with all relevant guidelines and regulations, including the Declaration of Helsinki and its later amendments. All participants gave written informed consent before taking part in the study.

### Stimuli and experimental design

The German vowels /a/, /e/, /i/, /o/, and /u/, recorded from an adult male native speaker, were used as the stimulus material in this study. Vowels were recorded in an anechoic room with 24-bit resolution and 48-kHz sampling rate, using a Brüel & Kjær condenser microphone (type 4193) and an RME Babyface audio interface. Each vowel was cut at zero-crossings right before vowel onset, cut to 800 ms length, equipped with a 50-ms Hann-windowed offset ramp, and high-pass filtered at 50 Hz (3^rd^-order Butterworth).

**Fig. 1:**
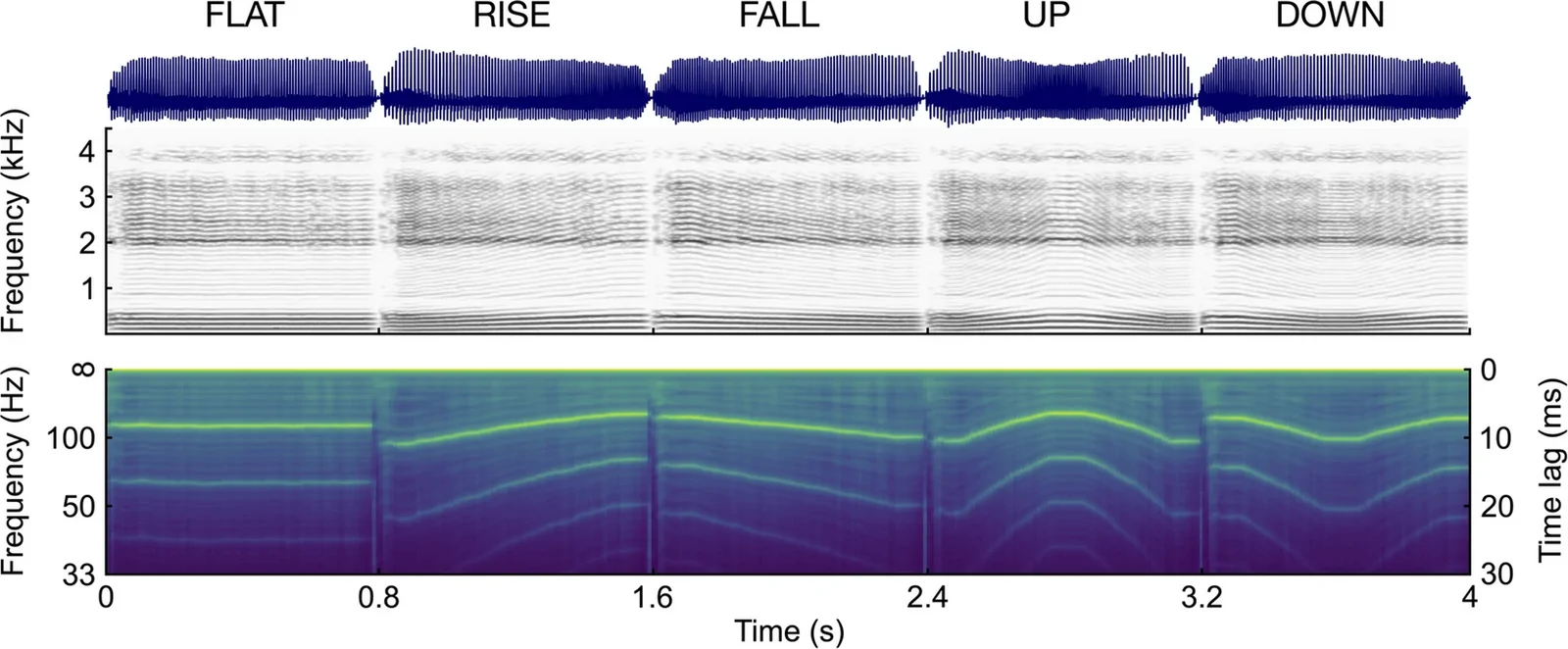
Illustration of the five prosodic contours including the vowel’s time waveform, a narrow-band spectrogram, and spectrographic representations of summary autocorrelation functions.

Next, five different prosodic contours (cf. Fig. 1) were created for each vowel using STRAIGHT^37^, a high-quality vocoder for independent manipulation of fundamental frequency (*f*_0_) and spectral information in speech sounds. For the first contour, flat, *f*_0_ was constant throughout the duration of the vowel; moreover, *f*_0_ was constant during the first and last 100 ms of *all* contours. For rise and fall, we introduced a 600 ms *f*_0_ glide of a major fifth that led to a rise or fall relative to the initial *f*_0_. up and down both consisted of a 250 ms upward or downward *f*_0_ glide (interval: major fifth), respectively, followed by a 100 ms static passage, and a 250 ms *f*_0_ glide back to the initial *f*_0_. All glides were linear in Hz. The vowels were then re-synthesized with the altered *f*_0_ information using STRAIGHT and normalized to a common root-mean-square (RMS) level. The narrow-band spectrograms presented in Fig. 1 show that the overall spectrum of the example vowel (/e/, mean *f*_0_ = 120 Hz) is very similar across the five prosodic contours, despite marked differences in its time waveform. To visualize the different prosodic contours of the single vowels, the lower part of Fig. 1 depicts spectrographic representations of summary autocorrelation functions^38,39^.

**Fig. 2:**
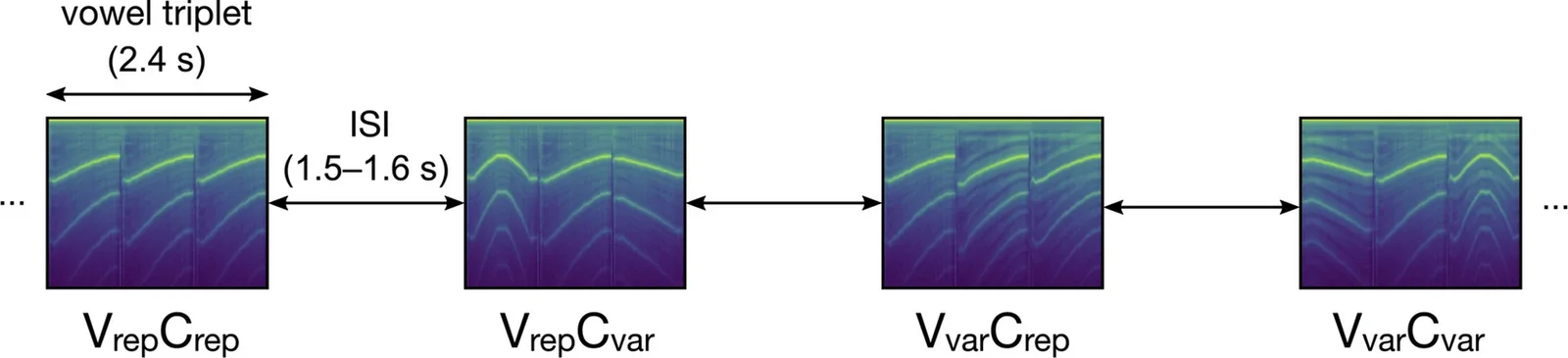
Example of the auditory stimulation including vowel triplets and ISI. V and C refer to triplets with repeated vs. varying vowel type and prosodic contour, respectively.

Afterwards, the single vowels were assembled into vowel triplets, following a 2*2 design in which the vowel type and/or the prosodic contour was repeated vs. varied between successive vowels within the triplet. (V_rep_ vs. V_var_: sequences with repeated vs. varying vowel type; C_rep_ vs. C_var_: repeated vs. varying prosodic contour sequences). In the sense of predictive coding, predictability was operationalized implicitly via the repetition structure of the stimulus sequences, rather than by explicit probability manipulations as in oddball paradigms: repeated sequences (i.e., V_rep_ or C_rep_) allow the formation of precise predictions about upcoming sound features, whereas varying sequences (i.e., V_var_ or C_var_) prevent reliable predictions. This also implies that in our experiment, predictability is defined at the local level of within-triplet regularities, instead of global stimulus probabilities. Figure 2 presents an example of the auditory stimulation; a more detailed description of the stimulation architecture is provided in the supplement (PART I).

Importantly, the nature of the stimulation implies that we distinguish between prosodic contour properties at two different levels. At the level of single vowels, prosody is classified according to five different contours (flat, rise, fall, up, and down), referring to how *f*_0_ evolves across time within the vowel. At the level of vowel triplets, the terms repeated and varying refer to whether vowel type or prosodic contour is repeated or varied across successive vowels within the triplet.

The stimulation was roved across mean *f*_0_ values of 80 Hz vs. 120 Hz to be independent of one specific *f*_0_ value; however, comparisons between *f*_0_ values were not of interest in our study. A total of 800 vowel triplets (i.e., 200 per sequence type) were delivered in pseudorandom order; they were designed such that all individual vowels, prosodic contours, and their respective transitions occurred equally often, balanced across within-triplet positions. As a result, each of the five single vowels was played 96 times with each of the five prosodic contours. The inter-stimulus interval (ISI) between the vowel triplets was randomly jittered between 1500–1600 ms, and the overall stimulation level was set to 70 dB SPL using a Brüel and Kjær measuring amplifier (Type 2610) and an ear simulator (Type 4157). The total duration of the MEG experiment was 53 minutes.

### Data acquisition

The participant’s AEF responses to the stimulation were recorded inside a magnetically shielded room (Imedco, Hägendorf, Switzerland), using a whole-head 122-channel gradiometer MEG system (Neuromag Elekta Oy, Helsinki, Finland,^40^) with 1000 Hz sampling rate and a 330 Hz lowpass filter. Prior to the recordings, the nasion, two pre-auricular points and 100 points distributed over the surface of the head were digitized as anatomical landmarks with a Polhemus, 3D Space, Isotrack2 system, individually for each participant. The auditory stimulation was presented via Etymotic Research (ER3) earphones with 90 cm plastic tubes and foam earpieces, using a 24-bit sound card (RME ADI 8DS AD/DA interface), an attenuator (Tucker-Davis Technologies PA-5) and a headphone buffer (Tucker Davis Technologies HB-7). During the recordings, the participants watched a silent movie of their own choice to maintain stable vigilance; they were instructed to direct their attention on the movie and ignore the auditory stimuli.

### Data analysis

In a first step, raw data were visually inspected and noisy channels were removed; then, episodes with amplitudes >8000 fT/cm or gradients >800 fT/cm/ms were automatically identified and excluded from further analyses, using the artifact rejection tool in the BESA 5.2 software package (BESA GmbH, Gräfelfing, Germany). Afterwards, the remaining responses to the single vowels were trigger-synchronously averaged into epochs from 200 ms before to 800 ms after vowel onset, separately for all 25 possible combinations of vowel types and prosodic contours; the baseline was defined as the mean amplitude in the 100 ms interval before vowel onset. In a second step, we also averaged the data to complete vowel triplets into epochs from 600 ms before to 3900 ms after triplet onset, with the baseline defined as the mean amplitude in the 100 ms interval before triplet onset; here, averaging was done separately for the four possible triplet types in which either vowel type and/or prosodic contour varied between successive vowels of the triplet.

**Fig. 3:**
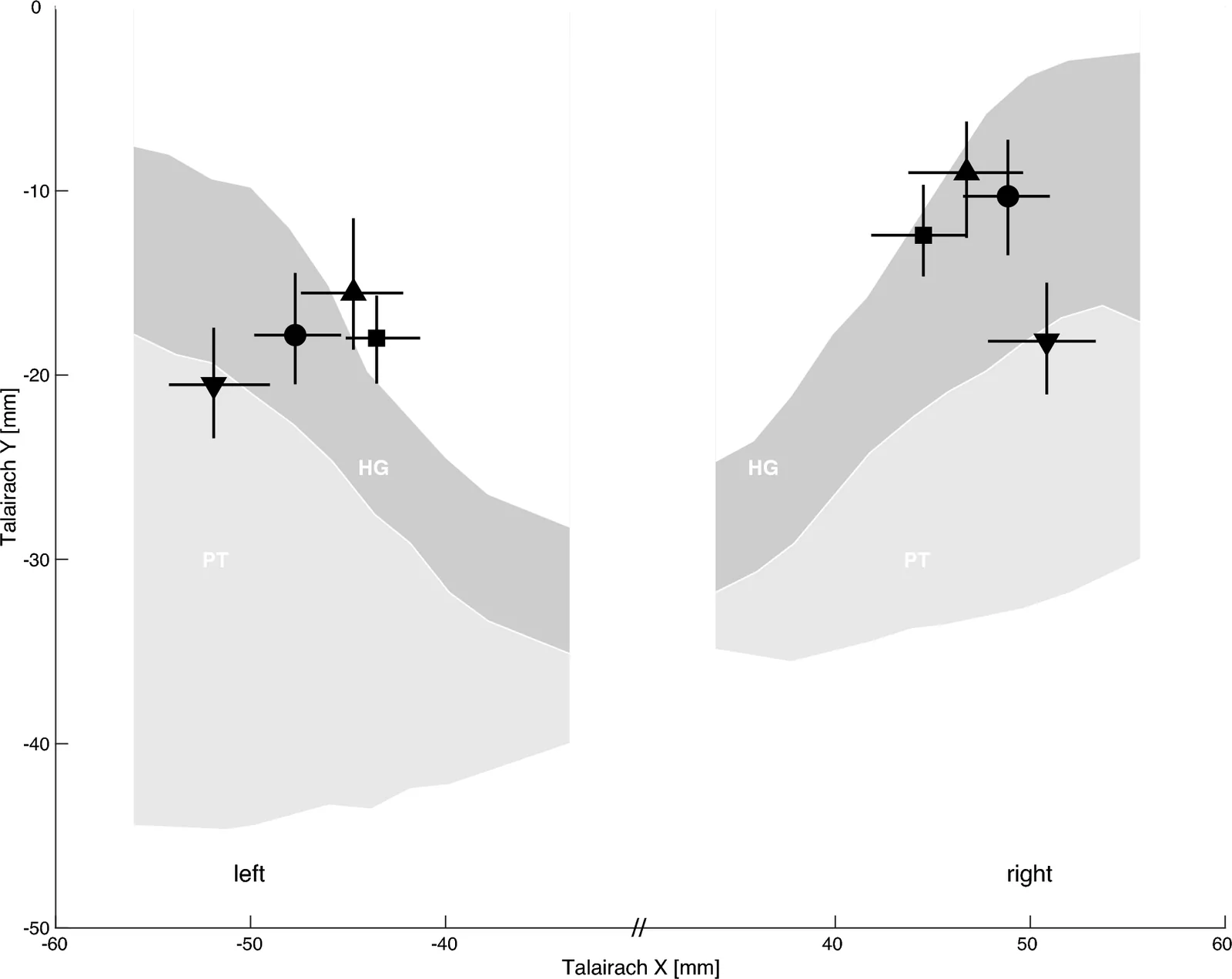
Dipole source locations, projected onto an axial map of the auditory cortex^46^; dark and light shadings illustrate Heschl’s gyrus (HG) and Planum temporale (PT), respectively. The average locations of the P1, N1, P2, and SF dipoles are shown with circles, downward triangles, upward triangles, and squares, respectively. Error bars represent 95% bias-corrected and accelerated bootstrap confidence intervals, based on 2,000 resamples^47^.

Subsequently, spatiotemporal source models^41–43^ with one dipole per hemisphere were fitted in BESA, individually for each participant, and separately for the P1, N1, P2, and SF components. All fits were based on the MEG data, pooled across all single vowels. Regarding the transient AEF responses, the fitting window was centered to a narrow time interval around the wave peak; for the SF, the last 300 ms of the vowels were used as fitting window. Moreover, when fitting the SF, a principal component analysis^44^ was also calculated over the last few milliseconds of the epoch to compensate for drift. Dipole fits were considered valid if source locations were in the range of |*x*| = 30–65 and *y* = -50–+10 according to the Talairach coordinate system^45^; where necessary, symmetry constraints were added to stabilize individual fits, but no further constraints were made regarding dipole position and orientation. Further information regarding the dipole models is summarized in Table 1; Figure 3 presents a projection of the dipole locations onto an axial map of the auditory cortex^46^. Finally, the models were applied as spatiotemporal filters to the MEG data, and the individual source waveforms of the participants were exported to MATLAB, separately for every condition (i.e., single vowel or vowel triplet).

**Table 1:** Dipole model information. For each dipole model, the table shows the bandpass filter settings during fitting (ZP, zero-phase; FW, forward), the number of participants for whom the fit was successful, and the number of participants in which a symmetry constraint was applied. The right part of the table shows the means and standard deviations for the source coordinates of the dipole models along the dimensions of the Talairach space^45^.

**AEF**	**Filter (Hz)**	**Successful**	**Symmetry**	**Left dipole**			**Right dipole**		
				***x***	***y***	***z***	***x***	***y***	***z***
**P1**	2–60 FP	23	3	-47.7 (5.3)	-17.9 (7.3)	4.0 (4.7)	48.9 (5.4)	-10.2 (7.5)	4.7 (5.8)
**N1**	2–20 FP	22	8	-51.9 (6.2)	-20.6 (7.3)	3.0 (5.2)	50.9 (6.7)	-18.1 (7.3)	2.4 (5.9)
**P2**	1–30 ZP	20	5	-44.7 (5.8)	-15.6 (7.8)	4.1 (7.4)	46.7 (6.8)	-9.0 (7.2)	4.0 (6.6)
**SF**	0–30 ZP	24	3	-43.5 (4.6)	-18.0 (6.1)	2.2 (4.9)	44.5 (6.0)	-12.4 (6.3)	3.0 (6.7)

Statistical analyses were accomplished with repeated-measures ANOVAs in SPSS 27.0 (IBM, Corp., Armonk, NY), including main effects, first/second order interactions, and appropriate Greenhouse-Geisser corrections, separately for the P1, N1, P2, and SF components; Hommel-adjusted post-hoc *t*-tests were calculated in MATLAB (The Mathworks, Inc., Natick, MA) when necessary. We followed a two-stage approach:

- Stage #1 of the analysis covered the responses to single vowels and included the factors HEMI (left vs. right), VOWEL_1_ (/a/, /e/, /i/, /o/, or /u/) and CONTOUR_1_ (flat, rise, fall, up, and down).
- Stage #2 of the analysis covered the complete vowel triplets and included the factors HEMI (left vs. right), VOWEL_2_ (triplets with repeated vs. varying vowel type, i.e., V_rep_ vs. V_var_), CONTOUR_2_ (repeated vs. varying prosodic contour sequences, i.e., C_rep_ vs. C_var_) and POS (1^st^, 2^nd^, or 3^rd^ position within the triplet).

ANOVAs were fed with the peak amplitudes and latencies of the transient AEF components; for the SF, mean amplitudes within the last 300 ms of the vowels were used. Transient peaks were automatically detected within their respective time windows using the min/max function in MATLAB, separately for each participant, condition, and dipole; the resulting values were visually double checked to ensure correct peak identification. Value extraction was done with the filter settings that had been applied during dipole fitting, with the exception of the SF in analysis stage #1 where a 0.15 Hz high-pass filter was added to ameliorate drift. Moreover, baselines were adjusted to the 100 ms interval before the respective epoch onset, except for the SF at the analysis stage #2 where the baseline always covered the 100 ms before triplet onset.

## Results

### Responses to single vowels (analysis stage #1)

**Fig. 4:**
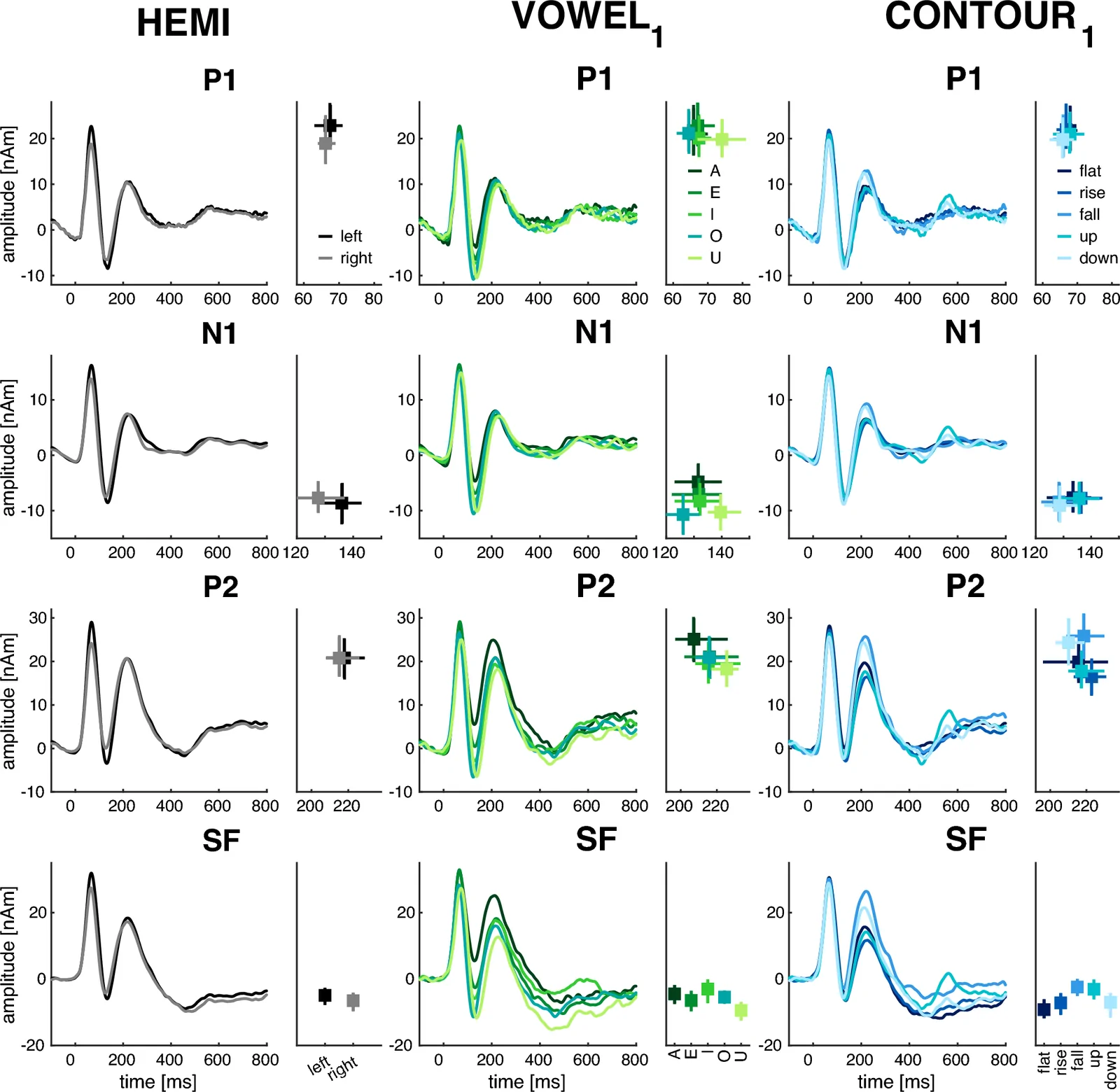
Neuromagnetic activity in response to the single vowels (analysis stage #1). For each dipole model (P1, N1, P2, and SF) and ANOVA main factor (HEMI, VOWEL_1_, and CONTOUR_1_), the figure presents the corresponding source waveforms in rows and columns, respectively, together with their 95% bias-corrected and accelerated bootstrap confidence intervals, based on 2,000 resamples^47^.

Figure 4 depicts the neuromagnetic activity in response to the single vowels, separately for each dipole model (P1, N1, P2, and SF; in rows) and ANOVA main factor (HEMI, VOWEL_1_, and CONTOUR_1_; in columns); the corresponding statistical results are presented in Table 2 (ANOVA) and in supplemental Tables 1 and 2 (post-hoc tests). Following stimulus onset, there was a tripartite AEF complex with prominent P1, N1, and P2 waves in all experimental conditions; this transient activity then evolved into a sustained field (SF) that persisted throughout the remaining stimulus duration. It is visible from the left-hand panels in Fig. 4 that, as a whole, the transient AEF components peaked somewhat earlier in the right than in the left hemisphere (cf. Table 2); moreover, the P1 wave in the right hemisphere had a smaller amplitude than that in the left hemisphere, whereas the SF was slightly larger in the right than in the left hemisphere (cf. Table 2).

**Table 2:** Statistical evaluation of the P1, N1, P2, and SF source waveforms in response to the single vowels (analysis stage #1), revealed by repeated-measures ANOVAs, presented separately for latency (lat) and amplitude (amp). *df*, degrees of freedom; *F*, test statistic; *p*, probability value; η^2^, partial squared eta. Statistically significant values are given in bold. * *p* < 0.05; ** *p* < 0.01; *** *p* < 0.001.

		**P1**		**N1**		**P2**		**SF**
		**Lat**	**Amp**	**Lat**	**Amp**	**Lat**	**Amp**	**Amp**
**VOWEL**_**1**_	***df***	**4, 88**	**4, 88**	**4, 84**	**4, 84**	**4, 76**	**4, 76**	**4, 92**
	***F***	**22.929**	**3.930**	**12.105**	**12.075**	**13.751**	**17.980**	**9.478**
	***p***	<0.001***	0.011*	<0.001***	<0.001***	<0.001***	<0.001***	<0.001***
	η^**2**^	**0.510**	**0.152**	**0.366**	**0.365**	**0.420**	**0.486**	**0.292**
**CONTOUR**_**1**_	***df***	4, 88	**4, 88**	**4, 84**	4, 84	**4, 76**	**4, 76**	**4, 92**
	***F***	2.116	**6.710**	**16.738**	1.545	**4.898**	**28.666**	**9.096**
	***p***	0.108	0.001**	<0.001***	0.221	0.004**	<0.001***	<0.001***
	η^**2**^	0.088	**0.234**	**0.444**	0.069	**0.205**	**0.601**	**0.283**
**HEMI**	***df***	**1, 22**	**1, 22**	**1, 21**	1, 21	**1, 19**	1, 19	**1, 23**
	***F***	**6.552**	**8.780**	**11.458**	0.273	**5.339**	0.587	**4.302**
	***p***	0.018*	0.007**	0.003**	0.607	0.032*	0.453	0.049*
	η^**2**^	**0.229**	**0.285**	**0.353**	0.013	**0.219**	0.030	**0.158**
**VOWEL**_**1**_ *CONTOUR_**1**_	***df***	16, 352	16, 352	16, 336	**16, 336**	16, 304	16, 304	16, 368
	***F***	0.901	1.521	1.124	**2.321**	0.861	1.550	0.948
	***p***	0.518	0.154	0.352	0.035*	0.536	0.154	0.477
	η^**2**^	0.039	0.065	0.051	**0.100**	0.043	0.075	0.040
**VOWEL**_**1**_ *HEMI	***df***	4, 88	4, 88	4, 84	4, 84	**4, 76**	4, 76	4, 92
	***F***	0.168	1.668	0.997	0.127	**3.355**	0.451	0.348
	***p***	0.892	0.188	0.402	0.916	0.025*	0.736	0.803
	η^**2**^	0.008	0.070	0.045	0.006	**0.015**	0.023	0.015
**CONTOUR**_**1**_ *HEMI	***df***	4, 88	**4, 88**	4, 84	4, 84	**4, 76**	4, 76	4, 92
	***F***	0.768	**3.396**	0.760	1.183	**3.830**	0.101	1.117
	***p***	0.532	0.017*	0.525	0.323	0.023*	0.974	0.351
	η^**2**^	0.034	**0.134**	0.035	0.053	**0.168**	0.005	0.046
**VOWEL**_**1**_ *CONTOUR_**1**_ *HEMI	***df***	16, 352	16, 352	16, 336	16, 336	16, 304	16, 304	16, 368
	***F***	0.856	1.747	1.108	1.258	0.768	1.626	0.430
	***p***	0.552	0.084	0.361	0.264	0.597	0.125	0.902
	η^**2**^	0.037	0.074	0.050	0.057	0.039	0.079	0.018

The middle panels of Fig. 4 show the neuromagnetic responses to the different vowels. The ANOVA results (cf. Table 2) and the pattern of post-hoc tests (cf. supplemental Tables 1 and 2) indicate that the vowel /u/ elicited transient waves with longer latency as compared to the other vowels, followed by a larger SF (cf. the bootstrap plots in the middle panels of Fig. 4). The vowel /o/ elicited larger N1 responses than the vowels /a/, /e/, and /i/; conversely, regarding the P2 wave, it was the vowel /a/ that elicited larger responses than the other vowels, and a significant VOWEL_1_*HEMI interaction revealed that the P2 response to the vowel /u/ appeared somewhat later in the left than in the right hemisphere (cf. supplemental Table 2).

An interesting result pattern occurred among the neural responses to the different prosodic contours (cf. right-hand panels of Fig. 4). The fall and down contours both led to particularly large P2 amplitudes (cf. supplemental Table 1); moreover, the SF turned out to be somewhat smaller following fall contours than in other conditions. A significant CONTOUR_1_*HEMI interaction revealed that the P2 response in the rise condition peaked later in the left than in the right hemisphere (cf. supplemental Table 2). There was also a weakly significant VOWEL_1_*CONTOUR_1_ interaction with respect to the N1 amplitude, but post-hoc tests were omitted for this effect due to the overly large number of pair-wise comparisons.

### Responses to vowel triplets (analysis stage #2)

**Fig. 5:**
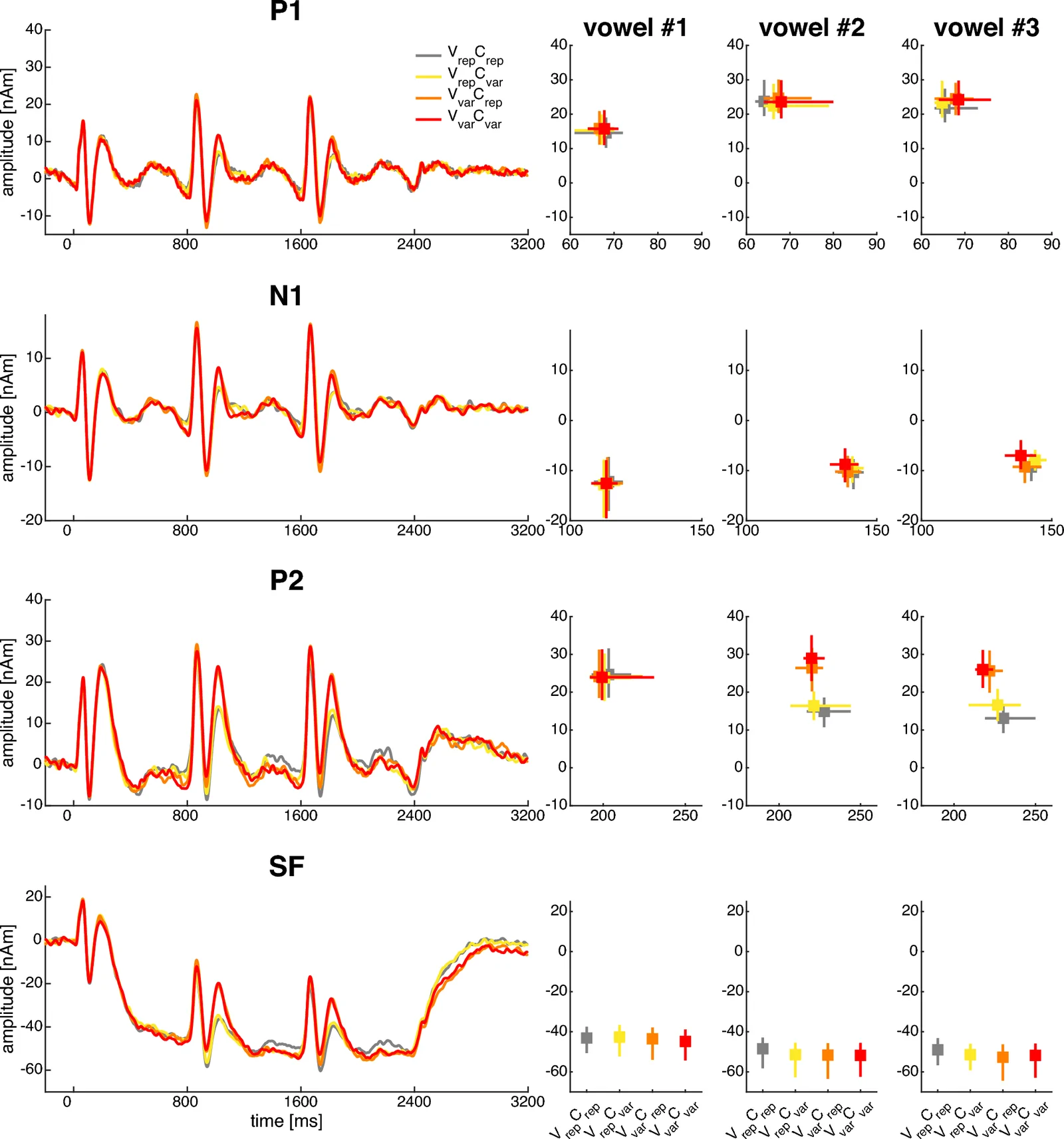
Neuromagnetic activity in response to the vowel triplets (analysis stage #2). The left panels of the figure present the source waveforms in response to the experimental conditions, separately for each dipole model (P1, N1, P2, and SF); the right panels of the figure show the corresponding peak latencies and amplitudes, separately for each single vowel in the triplet, together with their 95% bias-corrected and accelerated bootstrap confidence intervals, based on 2,000 resamples^47^. Please note that the right-hand panels are based on data, filtered and baseline-corrected as described in the Methods section; moreover, the time axes in these plots are expressed relative to the onset of the respective vowel.

The left panels of Fig. 5 show the source waveforms in response to the vowel triplets in the different experimental conditions, separately for each dipole model (P1, N1, P2, SF). The right panels of the figure present the corresponding peak latencies and amplitudes, separately for each single vowel in the triplet; the corresponding statistical results are denoted in Table 3 (ANOVA) and in supplemental Tables 3 and 4 (post-hoc tests). Each vowel within the triplet elicited a prominent transient P1-N1-P2 AEF complex, and there was also a large SF that evolved after the first vowel and lasted until the end of the triplet. The ANOVA results and the pattern of post-hoc tests indicate that the transient AEF waves peaked earlier after the first than after the subsequent vowels. Interestingly, the P1 amplitude was somewhat smaller in response to the first than after the second and third vowel; on the other hand, the N1, P2, and SF amplitudes appeared to decrease during the course of the vowel triplet (cf. right panels of Figure 5 and supplemental Table 3). According to two POS*HEMI interactions, this effect occurred predominantly in the right hemisphere in the N1 amplitude whereas in the SF, the effect was somewhat more pronounced in the left as compared to the right hemisphere (cf. supplemental Table 4). Regarding HEMI main effects, it turned out that the P1 amplitude was larger in the left hemisphere, and that the N1 latency was longer than in the right hemisphere.

**Table 3:** Statistical evaluation of the P1, N1, P2, and SF source waveforms in response to the vowel triplets (analysis stage #2), revealed by repeated-measures ANOVAs, presented separately for latency (lat) and amplitude (amp). *df*, degrees of freedom; *F*, test statistic; *p*, probability value; η^2^, partial squared eta. Statistically significant values are given in bold. * *p* < 0.05; ** *p* < 0.01; *** *p* < 0.001.

		**P1**		**N1**		**P2**		**SF**
		**Lat**	**Amp**	**Lat**	**Amp**	**Lat**	**Amp**	**Amp**
**VOWEL**_**2**_	***df***	1, 22	**1, 22**	1, 21	1, 21	1, 19	**1, 19**	1, 23
	***F***	3.382	**7.950**	3.016	0.885	1.388	**81.921**	2.356
	***p***	0.079	0.010*	0.097	0.358	0.253	<0.001***	0.138
	η^**2**^	0.133	**0.265**	0.126	0.040	0.068	**0.812**	0.093
**CONTOUR**_**2**_	***df***	**1, 22**	1, 22	1, 21	**1, 21**	1, 19	**1, 19**	1, 23
	***F***	**5.755**	0.238	3.238	**10.356**	3.503	**14.701**	2.810
	***p***	0.025*	0.631	0.086	0.004**	0.077	0.001**	0.107
	η^**2**^	**0.207**	0.011	0.134	**0.330**	0.156	**0.436**	0.109
**POS**	***df***	**2, 44**	**2, 44**	**2, 42**	**2, 42**	**2, 38**	**2, 38**	**2, 46**
	***F***	**12.997**	**44.851**	**47.900**	**8.681**	**13.407**	**5.834**	**39.027**
	***p***	0.001**	<0.001***	<0.001***	0.007**	<0.001***	0.018*	<0.001***
	η^**2**^	**0.371**	**0.671**	**0.695**	**0.292**	**0.414**	**0.235**	**0.629**
**HEMI**	***df***	1, 22	**1, 22**	**1, 21**	1, 21	1, 19	1, 19	1, 23
	***F***	2.334	**7.483**	**10.649**	0.235	4.275	0.495	0.010
	***P***	0.141	0.012*	0.004**	0.633	0.053	0.490	0.921
	η^**2**^	0.096	**0.254**	**0.336**	0.011	0.184	0.025	0.000
**VOWEL**_**2**_ *CONTOUR_**2**_	***df***	1, 22	1, 22	1, 21	1, 21	1, 19	1, 19	1, 23
	***F***	0.226	1.221	0.658	3.059	0.514	4.106	0.494
	***P***	0.639	0.281	0.426	0.095	0.482	0.057	0.489
	η^**2**^	0.010	0.053	0.030	0.127	0.026	0.178	0.021
**VOWEL**_**2**_ *POS	***df***	2, 44	2, 44	2, 42	2, 42	2, 38	**2, 38**	2, 46
	***F***	1.126	2.022	2.371	0.340	2.021	**30.324**	0.100
	***P***	0.330	0.156	0.117	0.685	0.159	<0.001***	0.885
	η^**2**^	0.049	0.084	0.101	0.016	0.096	**0.615**	0.004
**VOWEL**_**2**_ *HEMI	***df***	1, 22	**1, 22**	1, 21	1, 21	1, 19	1, 19	1, 23
	***F***	0.259	**9.953**	3.076	3.990	1.901	0.620	0.764
	***P***	0.616	0.005**	0.094	0.059	0.184	0.441	0.391
	η^**2**^	0.012	**0.311**	0.128	0.160	0.091	0.032	0.032
**CONTOUR**_**2**_ *POS	***df***	2, 44	2, 44	2, 42	**2, 42**	2, 38	2, 38	2, 46
	***F***	0.377	2.777	1.729	**4.491**	0.269	1.887	0.553
	***P***	0.658	0.076	0.197	0.025*	0.684	0.172	0.564
	η^**2**^	0.017	0.112	0.076	**0.176**	0.014	0.090	0.023
**CONTOUR**_**2**_ *HEMI	***df***	1, 22	1, 22	1, 21	1, 21	1, 19	1, 19	1, 23
	***F***	2.170	0.001	0.689	0.206	0.450	0.026	0.011
	***P***	0.155	0.979	0.416	0.655	0.511	0.873	0.917
	η^**2**^	0.090	0.000	0.032	0.010	0.023	0.001	0.000
**POS** *HEMI	***df***	2, 44	2, 44	2, 42	**2, 42**	2, 38	2, 38	**2, 46**
	***F***	1.143	0.574	1.208	**8.517**	0.674	0.150	**4.908**
	***P***	0.317	0.487	0.290	0.006**	0.505	0.803	0.020*
	η^**2**^	0.049	0.025	0.054	**0.289**	0.034	0.008	**0.176**
**VOWEL**_**2**_ *CONTOUR_**2**_ *POS	***df***	2, 44	2, 44	2, 42	2, 42	2, 38	**2, 38**	**2, 46**
	***F***	0.376	1.479	0.357	0.005	0.189	**8.883**	**3.402**
	***P***	0.624	0.241	0.663	0.993	0.814	0.001**	0.048*
	η^**2**^	0.017	0.063	0.017	0.000	0.010	**0.319**	**0.129**
**VOWEL**_**2**_ *CONTOUR_**2**_ *HEMI	***df***	1, 22	1, 22	1, 21	1, 21	1, 19	1, 19	1, 23
	***F***	1.145	0.146	2.251	1.131	0.116	0.828	0.106
	***P***	0.296	0.706	0.148	0.300	0.737	0.374	0.747
	η^**2**^	0.049	0.007	0.097	0.051	0.006	0.042	0.005
**VOWEL**_**2**_ *POS *HEMI	***df***	**2, 44**	2, 44	2, 42	2, 42	2, 38	2, 38	**2, 46**
	***F***	**6.997**	2.017	2.245	0.994	0.326	0.248	**6.316**
	***P***	0.003**	0.154	0.121	0.371	0.721	0.707	0.005**
	η^**2**^	**0.241**	0.084	0.097	0.045	0.017	0.013	**0.215**
**CONTOUR**_**2**_ *POS *HEMI	***df***	2, 44	2, 44	2, 42	2, 42	2, 38	2, 38	2, 46
	***F***	0.444	1.092	1.702	0.670	0.476	0.116	0.649
	***P***	0.640	0.336	0.201	0.510	0.585	0.877	0.508
	η^**2**^	0.020	0.047	0.075	0.031	0.024	0.006	0.027
**VOWEL**_**2**_ *CONTOUR_**2**_ *POS *HEMI	***df***	2, 44	2, 44	2, 42	2, 42	2, 38	2, 38	2, 46
	***F***	1.436	1.485	0.074	0.418	0.881	2.835	0.006
	***P***	0.249	0.238	0.871	0.612	0.417	0.089	0.992
	η^**2**^	0.061	0.063	0.004	0.020	0.044	0.130	0.000

There were also prominent differences between triplets with varying vs. repeated vowels. The combination of a VOWEL_2_ main effect and a VOWEL_2_*HEMI interaction revealed that the left-hemispheric P1 amplitude was larger in triplets with varying as compared to repeated vowels (cf. supplemental Table 4). Importantly, a VOWEL_2_ main effect and a VOWEL_2_*POS interaction conjointly showed that the P2 response to the second and third vowel only decreased in magnitude when the vowel was *not* varied within the triplet (cf. right panels of Figure 5 and supplemental Table 4).

A more complex result pattern occurred with respect to repeated vs. varying prosodic contour sequences. Here, a CONTOUR_2_ main effect and a CONTOUR_2_*POS interaction indicated that the N1 amplitude to the second and third vowel was somewhat *smaller* when the prosodic contour varied between the vowels in the triplet (cf. Figure 5 and supplemental Table 4). On the other hand, these conditions evoked larger P2 responses; and a CONTOUR_2_*VOWEL_2_*POS interaction revealed that for the third vowel, the P2 amplitude difference in response to repeated vs. varying prosodic contour sequences was pronounced when the vowel type was repeated throughout the triplet (cf. supplemental Table 4). Finally, P1 latencies were also somewhat longer in varying as compared to repeated prosodic contour sequences, and there were three more significant interaction effects (cf. Table 3), but corresponding post-hoc tests all missed significance (cf. supplemental Table 4).

## Discussion

The current MEG experiment was designed to investigate neuromagnetic correlates of prosodic contour information in vowel sequences. In summary, our data demonstrate that the P2 might be a prominent neural correlate of the representation of vowels as well as prosodic contours. Our results furthermore suggest that the P2 might be particularly sensitive to pitch glides. The corresponding source wave models were consistently robust, and their locations within the cortex resemble previous work.

### Neuronal activity in response to single vowels (analysis stage #1)

At the level of single vowels, the MEG results show that falling prosodic contours (fall and down) produce P2 responses with higher amplitudes and shorter latencies than rising (rise and up) and flat contours. We furthermore observed that the P2 is larger when vowel types and prosodic contours vary between subsequent vowels within the sequence. The current findings represent a valuable contribution to the literature since most earlier studies on pitch contour processing have used psychophysical paradigms^48–52^, while neurophysiological approaches are scarce. Furthermore, existing evidence appears inconsistent: Some authors reported enhanced sensitivity for downward frequency changes in psychophysical experiments^48^ or, on a neuronal level, in feline primary auditory cortex^53–55^; other studies, however, found evidence for enhanced upward frequency change responses, or no preference for any direction of pitch change^49–52,56–59^. The findings of the present work indicate differential neural sensitivity to prosodic contours at the level of early cortical AEF, but they do not imply a perceptual preference in the psychophysical sense. It would, however, be interesting to repeat the experiment with speakers of tonal languages (e.g., Mandarin) that use prosodic contours on a word and syllable level to convey information, to investigate how language expertise shapes the early neural representation of prosodic contours^60^.

While we did not observe significant effects in the post-hoc tests concerning the N1 response to different prosodic contours, our results suggest that the P2 might be particularly sensitive to pitch glides. This is in line with previous research: In his EEG study, Bidelman^21^ investigated the sensitivity of the pitch onset response (POR) in response to dynamic pitch contours (notably, the POR occurs within the time window of the N1 wave). Bidelman did not observe that the POR would distinguish rising from falling pitch contours. Its latency was, however, shorter in response to static as compared to dynamic pitch. Bidelman inferred from these findings that longer temporal integration windows might be required for dynamic signals such as pitch contours. In support of Bidelman’s notion, the current work emphasizes that the P2 shows pronounced modulation by prosodic contours while covering an appropriate time range. Further support for this idea comes from a recent MEG study by Andermann et al.^10^: In their study, the P2 response to within-sequence octave transitions appeared to indicate relative pitch. In contrast to our results, though, they also observed an N1 preference to upward as compared to downward pitch shifts.

Sound sequences composed of long, periodic stimuli are known to elicit large transient and sustained AEF (^8,10,61,62^), and this is also the case in the present experiment which employed vowels with a comparably long duration (800 ms). Such designs yield shallower pitch glides and frequency change rates than designs using brief vowels with rapid pitch transitions, and the prosodic modulation of the P2 wave observed in our study might, at least in part, be explained by this aspect. Clearly, while our findings are valid for the current design, they should not be assumed to generalize directly to naturally produced speech. However, higher stimulus rates and/or shorter sound durations will likely underappreciate the role of late responses in the cortical integration of pitch information; hence, long sounds may indeed pose a way to assess how the auditory system integrates such information across extended temporal windows. Generally speaking, studies on neural pitch processing exhibit substantial heterogeneity regarding the choice of both stimulus type (speech vs. non-speech) and stimulus parameters (e.g., pitch excursion or glide direction). These parameters critically determine temporal integration demands in auditory processing; accordingly, differences between our results and earlier findings are likely attributable to such choices, rather than pointing to an inconsistent neural representation of pitch contour.

Interestingly, analyses at the single vowel level revealed that the vowel /u/ elicited neural responses somewhat deviant from the effects in response to the other vowels. This observation nicely resembles earlier findings^63–65^. A look into formant maps of German vowels^66,67^ reveals that the first two formants of /u/ have particularly low energy maxima within the frequency spectrum, together with a small spectral distance between both formants. The latency of the N1 wave shows a high negative correlation with the frequency of a vowel’s first formant^65^; yet, similar relationships have also been reported for non-speech sounds^10,19,20^, suggesting that this effect reflects a general property of early auditory cortical processing rather than a language-specific phenomenon. The particularly low first and second formant frequencies of the vowel /u/, together with their reduced spectral separation, may therefore lead to delayed cortical responses, irrespective of linguistic context.

### Responses to vowel triplets (analysis stage #2)

At the triplet level, sequences in which the prosodic contour varied between successive vowels elicited P2 waves with higher amplitudes than repeated prosodic contour sequences, confirming our expectations and previous results^10^. The dependence of AEF response amplitudes on the repeated vs. varying nature of a prosodic contour sequence appears consistent with predictions formulated based on a predictive-coding framework. In short, this model proposes that auditory cortex uses incoming sounds to predict how the stimulation might continue^68,69^. Regarding repeated prosodic contour sequences, the current findings might be the result of neural adaptation (in the sense of repetition suppression) in the short-term context of highly predictable sound sequences^18,31,35^. However, when discussing neural adaptation, it should be noted that some studies suggest activity patterns that oppose our findings^70–73^: These results show that repetitive and therefore predictable stimuli elicit heightened neural activity, possibly to enable a focus on perceiving stable environmental stimuli^74^. As Andermann et al.^10^ noted, the studies mentioned above did mainly analyse MMN or P300 responses within oddball paradigms, instead of the transient and sustained responses to equiprobable stimuli which we examined. These earlier findings, therefore, cannot be directly compared to the current findings, and while the present results may appear compatible with the predictive coding framework as described above, they should not be interpreted as providing direct evidence for such mechanisms.

While varying prosodic contour sequences elicited strong P2 waves, we found that triplets in which the vowel type varied between successive stimuli produced P2 waves with even larger amplitudes. This amplitude difference might be explained when considering the language background of our participants, all native speakers of the non-tonal language German. Earlier research has shown that language experience shapes the early neural response to pitch^75–77^ and pitch contour^78,79^: Both speakers of tonal languages such as Mandarin Chinese and non-tonal languages such as English or German make use of prosodic contours to analyse incoming speech^80,81^. However, prosodic cues play a bigger role in tonal languages, providing in-depth linguistic information at the word level^82^. Perhaps consequentially, expertise in a tonal language may facilitate pitch perception^83^ and improve the ability to detect prosodic variations compared to experience in non-tonal languages only, as cross-language studies indicate^60,78^. With these findings in mind, one may speculate that the P2 activation pattern as observed in our MEG experiment might yield a divergent outcome in native tonal-language speakers, with comparably larger amplitudes in response to prosodic contour versus vowel transitions.

In our experiment, we did not observe SF response differences when comparing sequences with repeated vs. varying vowel type or prosodic contour. This finding contradicts the assumption whereby, parallel to the P2, sequences in which pitch varies between successive sounds elicit a larger SF than fixed pitch sequences. Interestingly, Andermann et al.^10^ reported similar findings for participants with low musical aptitude, but not for musically trained individuals, with the latter indeed showing a larger SF response to varying versus fixed pitch sequences. It is therefore possible that such an SF effect might only be visible in listeners with specific stimulus-related expertise, such as musicians or native speakers of tonal languages such as Mandarin Chinese. Several previous cross-language studies have revealed that tonal language natives outperform speakers of non-tonal languages in discriminating pitch contour changes in ongoing stimuli^60,78^. Repeating our current experiment with a different group of participants might reveal whether and how the SF differs in response to varying versus repeated prosodic contour sequences when presented to listeners with stimulus-related expertise.

In conclusion, our results suggest that the P2 plays an important role in the representation of vowels as well as prosodic contours; this might relate to the comparably longer time constants that are encompassed within the P2. Importantly, the present findings do not suggest that any specific AEF component represents a superior marker of pitch or prosody processing in the cortex; instead, the transient and sustained AEF appear to index distinct stages of the processing, from early acoustic feature encoding to the temporal integration of pitch contours. Conjointly assessing these components might lead to a more differentiated perspective on how pitch and prosody are represented in the auditory system.

## Supplementary Information

Supplementary Information.

## Data Availability

Raw data are available from the corresponding author upon reasonable request.
