# Supplementary material for: Neuromagnetic representation of vowel prosody in the auditory cortex
Source: Sci Rep. 2026 Sep 1;16:27410. doi: 10.1038/s41598-026-68466-x (PMC13534532; doi:10.1038/s41598-026-68466-x)
Supplement: Supplementary file 1 [file 41598_2026_68466_MOESM1_ESM.pdf]

Neuromagnetic representation of vowel prosody in the auditory cortex

Esther Megbel, Kurt Steinmetzger, André Rupp and Martin Andermann

+++ Supplementary Material +++

Part I: Details on the generation of the experimental stimulation

Part II: Supplemental Tables 1-4

PART I: Details on the generation of the experimental stimulation

- Our stimulus set comprises five different vowels (/a/, /e/, /i/, /o/, /u/).
- We define 10 ‘var’ sequences, each composed of three vowels. The vowels vary within the sequence, and a given vowel is only allowed to occur once within a sequence. Counted across the 10 ‘var’ sequences, each vowel occurs 6 times (twice at each position within the sequence), and each transition between two given vowels occurs once.
- Aside from the ‘var’ sequences, we also define 10 ‘rep’ sequences in which the vowel does not vary within the sequence (i.e., we have two identical ‘rep‘ sequences for each of the five vowels). There are no transitions between different vowels in the ‘rep’ sequences, but as with the ‘var’ sequences, each vowel occurs 6 times (twice at each position within the sequence).
- Here is an overview of the 10 ‘var’ and the 10 ‘rep’ sequences (numbers 1—5 refer to vowels /a/—/u/):

VAR	REP
1 – 2 – 4	1 – 1 – 1
2 – 3 – 5	1 – 1 – 1
3 – 4 – 1	2 – 2 – 2
4 – 5 – 2	2 – 2 – 2
5 – 1 – 3	3 – 3 – 3
4 – 2 – 1	3 – 3 – 3
5 – 3 – 2	4 – 4 – 4
1 – 4 – 3	4 – 4 – 4
2 – 5 – 4	5 – 5 – 5
3 – 1 – 5	5 – 5 – 5

- Next, we create 10 ‘var’ and 10 ‘rep’ sequences for the five different prosodic contours (FLAT, RISE, FALL, UP, DOWN), using exactly the same logic as outlined above for the vowel sequences.
- We now have 20 vowel sequences (10 rep, 10 var) and 20 prosodic contour sequences (10 rep, 10 var). We now combine each of the vowel sequences with each of the prosodic contour sequences. This gives us a total of 400 vowel triplets:
  - 100 in which both vowel type and prosodic contours are unchanged between successive stimuli
  - 100 in which only prosodic contour varies between successive stimuli (while vowel type is repeated)
  - 100 in which only vowel type varies between successive stimuli (while prosodic contour is repeated)
  - 100 in which both vowel type and prosodic contours are varied between successive stimuli
- For each of the 400 vowel triplets, we create two versions: one with mean f_0 = 80 Hz, and one with mean f_0 = 120 Hz. As a result, we have a total number of 800 vowel triplets in our stimulation; we play the vowel triplets in pseudorandom order.
- In terms of single vowels, each combination of a specific vowel type with a specific prosodic contour occurs 96 times during the stimulation (summed across both f_0 values).

PART II: Supplemental tables 1—4

Supplemental table 1. Posthoc tests for significant main effects of $VOWEL_1$ and $CONTOUR_1$ at stage #1 of the analysis (responses to single vowels). *df*, degrees of freedom; *t*, test statistic; *p*, Hommel-adjusted probability value. Significant comparisons are denoted in bold.

		**P1 (*df* = 22)**				**N1 (*df* = 21)**				**P2 (*df* = 19)**				**SF (*df* = 23)**	
		**latency**		**amplitude**		**latency**		**amplitude**		**latency**		**amplitude**		**amplitude**	
**effect**	**contrast**	**|*t*|**	***p***	**|*t*|**	***p***	**|*t*|**	***p***	**|*t*|**	***p***	**|*t*|**	***p***	**|*t*|**	***p***	**|*t*|**	***p***
**$VOWEL_1$**	/a/ vs. /e/	0.926	0.364	1.583	0.511	0.570	0.953	**3.060**	**0.030***	**3.884**	**0.007***	**4.639**	**0.001***	2.111	0.229
	/a/ vs. /i/	2.033	0.163	2.006	0.344	0.478	0.953	**3.669**	**0.010***	2.515	0.084	**5.123**	**0.001***	1.266	0.412
	/a/ vs. /o/	**3.290**	**0.020***	1.351	0.721	1.946	0.261	**4.599**	**0.001***	2.551	0.078	**5.812**	**<0.001***	1.022	0.412
	/a/ vs. /u/	**4.372**	**0.002***	1.668	0.476	**3.581**	**0.011***	**4.359**	**0.003***	**7.154**	**<0.001***	**7.691**	**<0.001***	**5.333**	**<0.001***
	/e/ vs. /i/	2.063	0.153	2.881	0.070	0.060	0.953	0.731	0.473	1.133	0.562	2.211	0.119	2.661	0.084
	/e/ vs. /o/	**2.914**	**0.040***	**3.117**	**0.045***	**3.868**	**0.006***	**3.549**	**0.013***	0.909	0.750	0.940	0.515	0.835	0.412
	/e/ vs. /u/	**6.337**	**<0.001***	2.686	0.108	**3.776**	**0.007***	2.699	0.054	**3.425**	**0.017***	**4.619**	**0.001***	**4.254**	**0.002***
	/i/ vs. /o/	1.567	0.263	0.923	0.721	2.805	0.053	**3.171**	**0.023***	0.066	0.948	2.125	0.141	1.724	0.393
	/i/ vs. /u/	**7.663**	**<0.001***	0.393	0.721	**3.612**	**0.010***	2.149	0.130	**4.276**	**0.003***	0.664	0.515	**5.464**	**<0.001***
	/o/ vs. /u/	**8.808**	**<0.001***	0.362	0.721	**7.805**	**<0.001***	1.388	0.359	**4.196**	**0.004***	2.730	0.067	**3.194**	**0.028***
**$CONTOUR_1$**	FLAT vs. RISE			2.465	0.154	0.167	0.967			2.393	0.224	**3.335**	**0.014***	1.902	0.209
	FLAT vs. FALL			2.364	0.191	1.593	0.630			0.131	0.897	**3.268**	**0.016***	**4.189**	**0.004***
	FLAT vs. UP			2.728	0.098	0.042	0.967			1.778	0.547	**3.152**	**0.021***	2.210	0.163
	FLAT vs. DOWN			2.972	0.063	2.557	0.147			0.899	0.897	1.743	0.293	0.015	0.988
	RISE vs. FALL			0.022	0.983	1.301	0.967			3.105	0.058	**4.833**	**<0.001***	1.952	0.195
	RISE vs. UP			0.703	0.983	0.189	0.967			0.949	0.897	0.014	0.989	0.373	0.988
	RISE vs. DOWN			0.575	0.983	1.910	0.441			2.094	0.349	**4.122**	**0.004***	1.727	0.293
	FALL vs. UP			0.853	0.983	2.873	0.082			1.655	0.604	**5.581**	**<0.001***	2.019	0.195
	FALL vs. DOWN			0.756	0.983	0.668	0.967			0.856	0.897	1.058	0.607	**3.757**	**0.009***
	UP vs. DOWN			0.311	0.983	**3.174**	**0.046***			0.513	0.897	**5.328**	**<0.001***	2.284	0.160

Supplemental table 2. Posthoc tests for significant interaction effects at stage #1 of the analysis (responses to single vowels). *df*, degrees of freedom; *t*, test statistic; *p*, Hommel-adjusted probability value. Significant comparisons are denoted in bold.

**effect**	**AEF (*df*)**	**contrast**	**|*t*|**	***p***
**$VOWEL_1$*HEMI**	P2 latency (19)	/a/: left vs. right	1.441	0.332
		/e/: left vs. right	1.855	0.238
		/i/: left vs. right	0.841	0.441
		/o/: left vs. right	2.168	0.159
		/u/: left vs. right	**3.090**	**0.030***
**$CONTOUR_1$*HEMI**	P1 amplitude (22)	FLAT: left vs. right	**3.477**	**0.011***
		RISE: left vs. right	**3.019**	**0.019***
		FALL: left vs. right	**2.193**	**0.039***
		UP: left vs. right	**2.578**	**0.034***
		DOWN: left vs. right	**3.044**	**0.018***
	P2 latency (19)	FLAT: left vs. right	0.715	0.484
		RISE: left vs. right	**3.198**	**0.024***
		FALL: left vs. right	1.418	0.345
		UP: left vs. right	1.922	0.230
		DOWN: left vs. right	1.424	0.342

Supplemental table 3. Posthoc tests for significant main effects of POS at stage #2 of the analysis (responses to vowel triplets). *df*, degrees of freedom; *t*, test statistic; *p*, Hommel-adjusted probability value. Significant comparisons are denoted in bold.

		**P1 (*df* = 22)**				**N1 (*df* = 21)**				**P2 (*df* = 19)**				**SF (*df* = 23)**	
		**latency**		**amplitude**		**latency**		**amplitude**		**latency**		**amplitude**		**amplitude**	
**effect**	**contrast**	**|*t*|**	***p***	**|*t*|**	***p***	**|*t*|**	***p***	**|*t*|**	***p***	**|*t*|**	***p***	**|*t*|**	***p***	**|*t*|**	***p***
**POS**	#1 vs. #2	**3.383**	**0.005***	**6.658**	**<0.001***	**6.650**	**<0.001***	**2.409**	**0.025***	**3.936**	**0.002***	2.115	0.096	**7.169**	**<0.001***
	#1 vs. #3	**4.182**	**0.001***	**7.279**	**<0.001***	**7.689**	**<0.001***	**3.392**	**0.006***	**3.841**	**0.002***	**2.760**	**0.037***	**6.281**	**<0.001***
	#2 vs. #3	0.513	0.613	0.083	0.935	1.467	0.157	**4.201**	**0.001***	0.911	0.374	1.702	0.105	0.506	0.618

Supplemental table 4. Posthoc tests for significant interaction effects at stage #2 of the analysis (responses to vowel triplets). V_{rep}/V_{var}, repeated vs. varying vowel sequences; C_{rep}/C_{var}, repeated vs. varying prosodic contours; *df*, degrees of freedom; *t*, test statistic; *p*, Hommel-adjusted probability value. Significant comparisons are denoted in bold.

effect	AEF (*df*)	contrast	|*t*|	*p*
**POS*HEMI**	N1 amplitude (21)	left: #1 vs. #2	1.208	0.241
		left: #1 vs. #3	2.178	0.082
		left: #2 vs. #3	**3.598**	**0.006***
		right: #1 vs. #2	**3.341**	**0.009***
		right: #1 vs. #3	**4.068**	**0.003***
		right: #2 vs. #3	**3.515**	**0.006***
	SF amplitude (23)	left: #1 vs. #2	**7.724**	**<0.001***
		left: #1 vs. #3	**6.109**	**<0.001***
		left: #2 vs. #3	0.076	0.940
		right: #1 vs. #2	**4.920**	**<0.001***
		right: #1 vs. #3	**4.357**	**0.001***
		right: #2 vs. #3	1.188	0.494
**POS*VOWEL$_2$**	P2 amplitude (19)	#1: V_{rep} vs. V_{var}	1.230	0.234
		#2: V_{rep} vs. V_{var}	**8.771**	**<0.001***
		#3: V_{rep} vs. V_{var}	**6.872**	**<0.001***
**POS*CONTOUR$_2$**	N1 amplitude (21)	#1: C_{rep} vs. C_{var}	0.021	0.984
		#2: C_{rep} vs. C_{var}	**3.010**	**0.013***
		#3: C_{rep} vs. C_{var}	**3.347**	**0.009***
**VOWEL$_2$*HEMI**	P1 amplitude (22)	left: V_{rep} vs. V_{var}	**3.369**	**0.006***
		right: V_{rep} vs. V_{var}	1.641	0.115
**VOWEL$_2$*CONTOUR$_2$*POS**	P2 amplitude (19)	#1 V_{rep}: C_{rep} vs. C_{var}	0.567	0.646
		#1 V_{var}: C_{rep} vs. C_{var}	0.885	0.646
		#2 V_{rep}: C_{rep} vs. C_{var}	2.520	0.104
		#2 V_{var}: C_{rep} vs. C_{var}	1.955	0.262
		#3 V_{rep}: C_{rep} vs. C_{var}	**4.214**	**0.003***
		#3 V_{var}: C_{rep} vs. C_{var}	0.467	0.646
	SF amplitude (23)	#1 V_{rep}: C_{rep} vs. C_{var}	0.285	0.984
		#1 V_{var}: C_{rep} vs. C_{var}	0.698	0.984
		#2 V_{rep}: C_{rep} vs. C_{var}	2.009	0.282
		#2 V_{var}: C_{rep} vs. C_{var}	0.020	0.984
		#3 V_{rep}: C_{rep} vs. C_{var}	1.865	0.375
		#3 V_{var}: C_{rep} vs. C_{var}	0.678	0.984
**VOWEL$_2$*HEMI*POS**	P1 latency (22)	#1 left: V_{rep} vs. V_{var}	0.140	0.890
		#1 right: V_{rep} vs. V_{var}	0.841	0.890
		#2 left: V_{rep} vs. V_{var}	2.485	0.105
		#2 right: V_{rep} vs. V_{var}	0.162	0.890
		#3 left: V_{rep} vs. V_{var}	1.049	0.890
		#3 right: V_{rep} vs. V_{var}	2.325	0.149
	SF amplitude (23)	#1 left: V_{rep} vs. V_{var}	0.405	0.875
		#1 right: V_{rep} vs. V_{var}	1.421	0.675
		#2 left: V_{rep} vs. V_{var}	1.228	0.875
		#2 right: V_{rep} vs. V_{var}	0.159	0.875
		#3 left: V_{rep} vs. V_{var}	1.765	0.464
		#3 right: V_{rep} vs. V_{var}	0.428	0.875
